# Circ_0000052/miR‐382‐3p axis induces PD‐L1 expression and regulates cell proliferation and immune evasion in head and neck squamous cell carcinoma

**DOI:** 10.1111/jcmm.17643

**Published:** 2022-12-14

**Authors:** De‐Jun Zhang, Ze‐Ming Fu, Ying‐Yuan Guo, Fang Guo, Yi‐Ning Wan, Guo‐Fang Guan

**Affiliations:** ^1^ Department of Otolaryngology‐Head and Neck Surgery The Second Hospital of Jilin University Changchun China

**Keywords:** circular RNA, head and neck squamous cell carcinoma, immunotherapy, microRNA, PD‐L1

## Abstract

A better understanding of the mechanisms underlying PD‐L1 aberrant expression in head and neck squamous cell carcinoma (HNSCC) will help reveal predictive biomarkers and overcome resistance to treatment. In this study, the prognostic significance of PD‐L1 in forty‐five HNSCC archival samples was determined by qRT‐PCR. The biological function associated with malignant behaviour was assessed by PD‐L1 depletion, miR‐382‐3p re‐expression and regulation of circ_0000052. The interactions of PD‐L1‐miRNA and miRNA‐circRNA were determined by qRT‐PCR, Western blot analysis, dual‐luciferase reporter assays and RNA immunoprecipitation assays. PD‐L1 was highly expressed in patient samples and cancer cell lines. Higher levels of PD‐L1 were associated with patient recurrences and play a pivotal role in regulating cell proliferation, migration, invasion, clonogenicity and apoptosis. In addition to demonstrating that the IFN‐γ/JAK2/STAT1 signalling pathway can induce PD‐L1 overexpression in HNSCC, a novel mechanism by which upregulated circ_0000052 mediates PD‐L1 overexpression was also demonstrated. To do this, circ_0000052 competitively binds to miR‐382‐3p and alleviates its repression of PD‐L1. This leads to overexpression of PD‐L1, causing the aggressiveness of the cells. Our data demonstrate that circ_0000052 is oncogenic, and the circ_0000052/miR‐382‐3p/PD‐L1 axis is critical in HNSCC progression. The manipulation of circRNAs/miRNAs in combination with anti‐PD‐L1 therapy may improve personalized disease management.

## 1 INTRODUCTION

Head and neck squamous cell carcinoma (HNSCC), which includes tumours of the lip, oral cavity, larynx, oropharynx and hypopharynx, accounted for 3.9% of new cases and 3.7% of cancer deaths in 2020. In the Asia region, it has been observed to have the highest incidence.^1^ Despite multimodal treatment, including surgery and radiotherapy, with or without conventional chemotherapy, around 30%–40% of patients with aggressive disease develop distant metastases within 5 years.^2^ Checkpoint blockade immunotherapy has achieved significant efficacy in improving clinical outcomes by increasing antitumor immune responses.^3^ However, only a small percentage of patients are in remission. Therefore, investigating the underlying mechanisms associated with immunotherapy may help overcome some of the barriers and improve treatment options for HNSCC.

Recent advances in oncology have demonstrated that analysing the multiplex relationships between tumour cells and their adjacent tumour microenvironments (TME) is critical for understanding tumour immune escape mechanisms.^4^ Increasing evidence suggests that PD‐1 (programmed cell death‐1), as an important immune checkpoint molecule, is mainly expressed on immune cells and negatively regulates antigen‐specific T‐cell immunity by binding to its receptor PD‐L1 (programmed cell death‐1), acting as immunosuppressive and contributing to cancer evasion.^5^ PD‐L1 (CD274 gene) is upregulated in many cell types, including cancer cells, in response to pro‐inflammatory cytokines. PD‐L1 is expressed at different levels in various cancers and is associated with poor prognosis in melanoma,^6^ NSCLC^7^ and colorectal cancer.^8^ However, the clinical implications of PD‐L1 expression vary across studies. The Pembrolizumab trial found that patients with ≥1% PD‐L1 expression had significantly better survival.^9^ In contrast, in some studies, PD‐L1‐negative tumours also responded to treatment, and most patients with solid tumours persisted with primary or acquired resistance,^10^ suggesting the existence of other mechanisms, the study of which will help guide treatment selection.

Multiple microRNAs (miRNAs) that bind to the PD‐L1 3′UTR can regulate its expression. Numerous miRNAs have been reported to be involved in the regulation of HNSCC. Overexpression of let‐7a/b directly reduces PD‐L1 expression by binding to its 3'UTR, thereby stimulating antitumor immunity and inhibiting HNSCC growth in vivo.^11^ The upregulated miR‐21 inhibits activation of STAT3 resulting in the suppression of lymphocyte migration, indirectly regulating PD‐L1 expression.^12^ Recent studies have also shown that aberrant expression of circular RNAs (circRNAs) is closely related to cancer development. CircRNAs can act as competitive endogenous RNA sponges to bind miRNAs and regulate their transcription.^13^ Circ‐CPA4 regulates drug resistance in NSCLC cells through the let‐7 miRNA/PD‐L1 axis.^14^ However, the functions of most circRNAs in HNSCC remain largely unknown. In the current study, we propose a molecular mechanism whereby abnormal overexpression of circ_0000052 sponges miR‐382‐3p, which releases the inhibition of PD‐L1, resulting in the upregulation of PD‐L1 in HNSCC. Our work will broaden our knowledge of the pathogenesis of HNSCC and provide potential therapeutic targets for the management of the disease.

## 2 METHODS

### 2.1 Patient materials and cell lines

Between 2008 and 2019, forty‐five diagnostic formalin‐fixed paraffin‐embedded (FFPE) samples were collected from patients with head and neck squamous cell carcinoma (HNSCC) at the Second Hospital of Jilin University in Changchun, China. All studied tumour specimens were confirmed on haematoxylin‐and‐eosin‐stained slides using pathologically standard diagnostic criteria, and only samples containing >60% tumour cells were considered for the study (Table 1). An additional 15 adjacent normal tissue samples were collected as controls. Subsequently, from the 45 samples, 38 available samples were used to detect the expression of miR‐382‐3p and circ_0000052. This study was approved by the Medical Ethics Committee of Jilin University (no. 2021–157).

**TABLE 1:** Patient's characteristics

Characteristics	No. of patients (%)
Age, years	
Median	53.2
Range	37–76.8
Sex	
Men	35 (77.7)
Women	10 (22.3)
Smoking status	
No or light	17 (37.7)
Heavy	28 (62.3)
T classification	
T1	10 (22.2)
T2	14 (31.1)
T3/4	21 (46.7)
N classification	
N0/N1	11 (24.4)
N2/3	34 (75.6)
Clinical Stage	
I/II	12 (26.7)
III/IV	33 (73.3)
RT intent	
RT	35 (77.8)
RT‐CRT	10 (22.2)

Abbreviations: RT, radiotherapy; RT‐CRT, radiotherapy combined with chemotherapy.

Two human head and neck squamous cell cancer cell lines, FaDu (human hypopharyngeal squamous cancer) and SCC‐9 (human tongue squamous cell carcinoma), obtained from the American Type Culture Collection, and NOE cells (normal oral epithelial cells), from Celprogen (San Pedro, CA), were maintained in DMEM with 10% foetal bovine serum (FBS). All cells were maintained in a 37°C incubator with a humidified 5% of CO_2_ and tested to be free from mycoplasma contamination.

### 2.2 RNA extraction and quantification of PD‐L1 and miRNAs

A total of 5–10 (8‐μm) sections were obtained from each sample, and total RNA was extracted from the samples using the Recover All Total Nucleic Acid Isolation kit for FFPE (Thermo Fisher Scientific, Inc.) or from cell lines using the RNA extraction kit from Qiagen according to the manufacturer's instructions. The SuperScript III Reverse Transcriptase (Invitrogen) was used for RNA reverse transcription. Quantitative real‐time PCR analysis was performed using SYBR Green PCR Master Mix (Thermo Fisher Scientific, Inc.) and the ABI PRISM 7900 Sequence Detection System (Applied Biosystems). GAPDH was used as an endogenous control.

The expression of miRNAs was measured using the standard Taqman MicroRNA assay (Applied Biosystems). RNA was reverse‐transcribed with a MultiScribe reverse transcriptase using a stem‐loop RT primer designed to specifically hybridize with an individual miRNA. The RT products were subsequently amplified with sequence‐specific primers. RNU6 was used as an endogenous control. Table S1 details the primers used in the study, including those for qRT‐PCR.

### 2.3 RNase R treatment and quantification of circRNAs

Total RNA was treated with or without 3 U/μg RNase R (Geneseed Biotech Co., Ltd.) to remove genomic DNA, then purified using a RNeasy MinElute Cleanup kit (Qiagen). The stability of circRNAs was also measured by qRT‐PCR after adding 2 μg/ml transcription inhibitor of actinomycin D or DMSO (Sigma‐Aldrich) as a control.

### 2.4 Transient transfections and proliferation assays

RNA interference experiments were used to evaluate the biological effects of PD‐L1 in vitro. Two PD‐L1‐target siRNAs (siRNA1 and siRNA2), siRNA to Stat1, premiR‐382‐3p, antimiR‐382‐3p, premiR‐375‐5p, the negative control siRNA (NC), the mock mimic miRNA negative control (MC) and antimiRNA controls were purchased from Thermo Fisher Scientific (Shanghai, China). The pLV‐cir vectors carrying the overexpressing circ_0000052 plasmids were designed and synthesized by GenePharma (Shanghai, China).

HNSCC cells were seeded into 96‐well plates or 6‐well plates and transfected using the LipofectAMINE RNAiMAX (Life Technologies) protocol. The cytopathic effects of Fadu and SCC‐9 cells post‐transfection were evaluated using the CellTiter 96 Non‐Radioactive Cell Proliferation Assay (MTS) (Promega Bio Sciences). Cell proliferative activity was measured at 24, 48 and 72 h after transfection.

### 2.5 Cell migration and invasion assays

The cellular effects of knockdown of PD‐L1 were further investigated in FaDu and SCC‐9 cells using the BD Biosciences BioCoat control chamber and Matrigel invasion chamber. 1 × 10^5^ cells were transfected with siRNA1/siRNA2 or siRNA scramble control, or si‐circ_0000052 or si‐circ negative control, and plated on either the 6‐well control inserts (PET membrane) or trans‐well chambers pre‐coated with Matrigel. A medium containing 15% foetal bovine serum in the lower chamber served as the chemoattractant. After 24 h incubation, non‐migrating or invading cells were removed from the upper surface of the membrane with cotton swabs. The migrating or invasive cells attached to the low surface of the membrane insert were then fixed and stained with Diff‐Quick Stain (BD Biosciences). The number of migrating or invasive cells was counted under a microscope.

### 2.6 Cell apoptosis and colony formation assays

Caspase‐Glo 3/7 assay kit (Promega Corp.) used for assessing enzyme activity. Cells were transfected with siRNA1‐PD‐L1 or control siRNA (40 nM). The activities of caspase‐3/7 were measured at 24 and 48 h post‐transfection. The transfected cells were added to reaction buffer and DEVD‐p‐NA substrate, after incubation at 37°C, the caspase activity was analysed with a microplate reader. For the colony formation assay, cells were transfected in 12‐well plates. At 48 h after, cells were harvested, and 300 or 500 cells were re‐seeded onto 6‐well plates in triplicate. After 2 weeks in culture, the plates were fixed and stained, and the number of colonies was counted using a microscope. The percentage of surviving cells was calculated by comparing them to transfected cells or scramble control (SC) cells.

### 2.7 Western blotting

The cells were treated with IFN‐ or transfected with siPD‐L1s, pre‐ or anti‐miRs, or si_circRNAs. At 72 h after, the cells were collected and lysed. Protein extracts were prepared and quantified using the BCA method. 20 μg of protein was loaded onto 10% Tris‐glycine protein gels and then transferred onto a nitrocellulose membrane. After blocking with 5% milk in Tris‐buffered saline with 0.1% Tween‐20 (TBST), the membranes were probed with rabbit anti‐PD‐L1 (ab213524, Abcam, USA) and mouse anti‐beta Actin (ab8226, Abcam, USA) antibodies overnight, followed by incubation with the second antibodies (Abcam, USA) labelled with horseradish peroxidase for 2 h. Signals were visualized using the ECL Western blotting substrate system.

### 2.8 Luciferase reporter assay

The interactions between miRNAs and PD‐L1 or circRNAs with miRNAs were assessed by using the Dual‐report luciferase assays. The sequences of wild‐type or mutant PD‐L1 3'‐UTR regions, which contained miR‐382‐3p or miR‐375‐5p putative binding sites, were amplified by PCR, respectively. Similarly, the sequence of the wild‐type or mutant circ_0000052, which contains the binding site for miR‐382‐3p, was also amplified. After inserting the products into pmiRreport luciferase vectors (Ambion), we co‐transfected those vectors with miR‐382‐3p mimics or miR‐375‐5p mimics, or scramble control in cells. The direct interaction between the 3'UTR of PD‐L1 or circ_0000052 and miR‐382‐3p was detected by co‐transfection of miRNA mimics in Fadu or SCC‐9 cells. A pRL‐SV40 vector (Promega) containing Renilla luciferase was also transfected to each well as a reference control. At 48 h post‐transfection, cells were harvested, and Dual‐Glo luciferase assay system (Promega) was used to assess both Firefly and Renilla luciferase activities.

### 2.9 Prediction of PD‐L1 and RNA interaction

Gene target prediction softwares (miRwalk, miRDB, miRSearch, miRSystem and MicroT‐CDS) were used to predict microRNAs that may potentially target PD‐L1. Based on the analysis of the Circular RNA Interactome (https://circinteractome.nia.nih.gov/) and TargetscanHuman 7.2 (http://www.targetscan.org/vert_72/), the interaction between circ_0000052 (AGO1) and miRNAs was predicted.

### 2.10 RNA immunoprecipitation (RIP) assay

According to the instructions of the Magna RIP™ kit (Millipore, USA), the transfected cells (premiR‐382‐3p or negative control) were lysed in RIP lysis buffer. The cell extract was then incubated with magnetic beads coated with anti‐argonaute2 (AGO2) antibodies. At a magnetic perforation of 4°C for 6 h, anti‐immunoglobulin G (Anti‐IgG) was used as a negative control. The beads were washed, and the protein was removed using proteinase K (Sigma) from magnetic beads. After RNA isolation, the levels of circ_0000052 were then measured.

### 2.11 Fluorescence in situ hybridization (FISH)

Cancer cells were seeded on chamber slides. After fixation with 4% paraformaldehyde and blocking with 3% H_2_O_2_, the cells were incubated with Cy3‐conjugated circ_0000052 probes (LMAI Bio, Shanghai, China) for 24 h at 4°C. DAPI was used for cell nucleus staining. The images were analysed using confocal AxioImager microscopy (Zeiss Inc).

### 2.12 Animal experiments

shRNA (short hairpin RNA) against circ_0000052 and the negative control shRNA (shNC) were designed and purchased from GenePharma (Shanghai, China). The shRNA plasmids, pGPU6/Hygro and pGPH1 were co‐transfected into HEK‐293 T cells. The supernatant containing the lentivirus particles was used to infect Fadu cells and selected by puromycin (2 mg/mL). Nine (6–8 weeks old) mice with severe combined immunodeficiency (SCID) were divided into three groups (*n* = 3 each): negative control (NC), transfected premiR‐383‐3p, and shcirc_0000052. 48 h after transfection, 1 × 10^7^ cells were mixed with 100 μl of matrigel and injected into the right forelimb of the mice intramuscularly. Tumour growth was measured twice per week. Tumour volume was calculated using the formula: V = (Width × 2 × Length)/2.^15^ All the animal experiments were approved by the Institutional Animal Care and Use Committee‐IACUC of Jilin University (No. SY202210015).

### 2.13 Statistical analysis

The Kaplan–Meier analysis was conducted to depict the overall survival probability, and the log‐rank test was employed for comparison. All data were expressed as mean ± SE; *p* values were two‐sided, with α = 0.05. All analyses were performed using SAS 9.4 (SAS Institute). Graphs were constructed using GraphPad Prism software.

## 3 RESULTS

### 3.1 PD‐L1 is frequently expressed in HNSCC and is associated with poor prognosis

**FIGURE 1:**
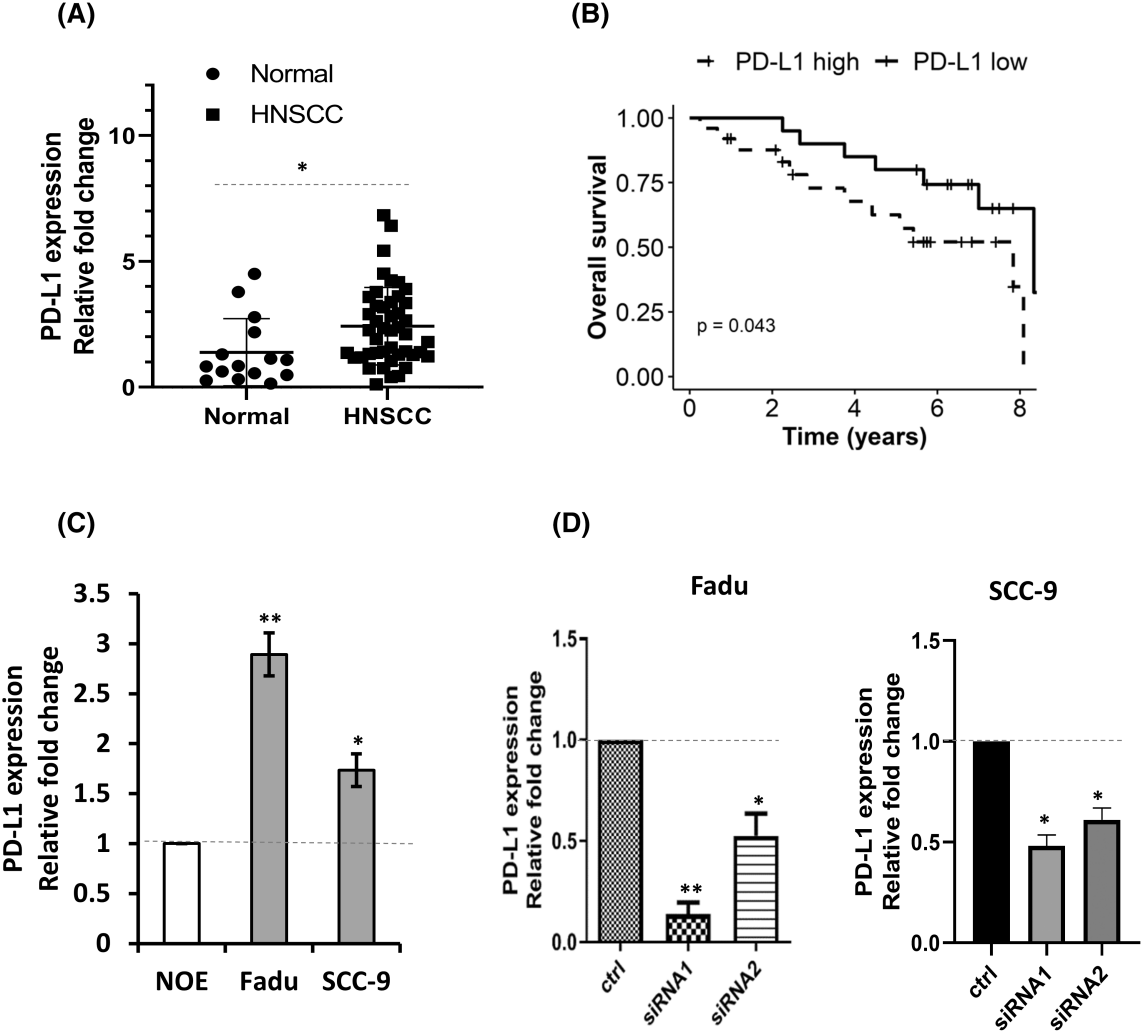
PD‐L1 overexpression is associated with poor prognosis in HNSCC patients. (A) The expression of PD‐L1 was increased in HNSCC examined by RT‐qPCR. (B) Higher PD‐L1 expression was associated with worse overall survival as analysed by Kaplan–Meier survival analysis and the log‐rank test (*p* < 0.05). (C) PD‐L1 overexpression in two HNSCC cell lines. (D) siRNAs effectively deplete PD‐L1. * *p* < 0.05; ** *p* < 0.01.

The clinical status of PD‐L1 in HNSCC was analysed by qRT‐PCR. Expression levels of PD‐L1 were evaluated in 45 cancer specimens and 15 adjacent normal tissues. The majority of cancer tissues exhibited higher levels of PD‐L1 expression compared with adjacent normal tissues (Figure 1A, *p* = 0.05). Kaplan–Meier survival analysis showed that patients with higher PD‐L1 expression had worse overall survival (OS) compared with patients with lower PD‐L1 expression (Figure 1B, *p* = 0.043). These findings implicate that PD‐L1 is involved in the progression of HNSCC and lead us to investigate whether silencing of PD‐L1 can functionally alter the malignant behaviour of tumour cells. PD‐L1 expression was then examined in two HNSCC cancer cell lines: Fadu and SCC‐9. By comparison with the normal oral epithelial cell line NOE, PD‐L1 was significantly upregulated in cancer cells (Figure 1C, *p* < 0.01). Two siRNAs specific against PD‐L1 were transfected into Fadu and SCC‐9 cells. Of note, the levels of PD‐L1 were reduced significantly to 20% of siRNA1 and 50% of siRNA2 at 48 h after transfection, and showed a similar pattern of reduction in SCC‐9 cells (Figure 1D, *p* < 0.05). In accordance with the data above, these reductions were further confirmed by Western blot analysis (Figure S1).

### 3.2 Effects of PD‐L1 silencing on cell proliferation, migration, invasion and apoptosis

**FIGURE 2:**
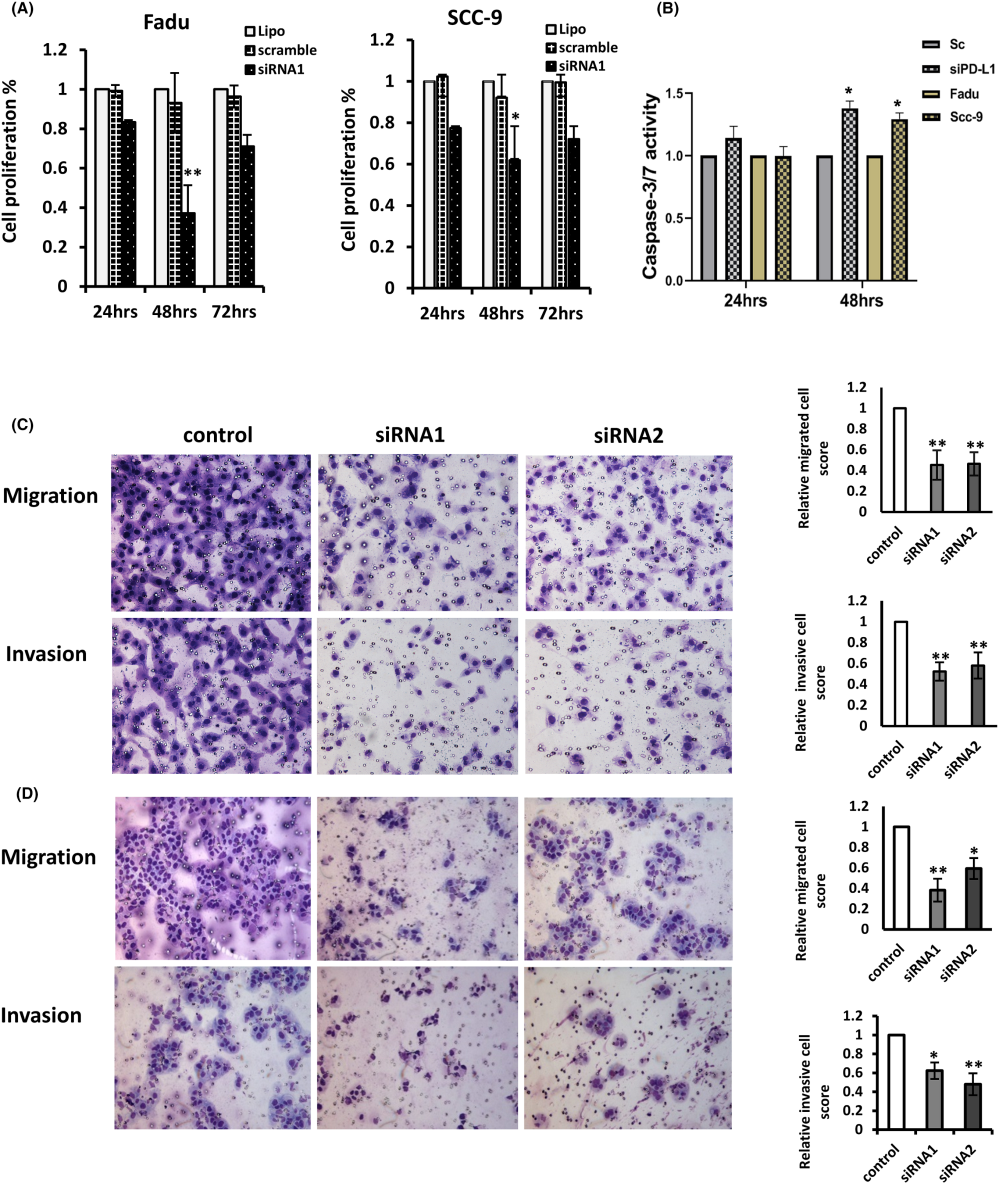
PD‐L1 depletion reduces cell proliferation, migration and invasion while increasing apoptosis activity. (A) siRNA for PD‐L1 was transfected into Fadu and SCC‐9 cells, respectively, and cell viability was determined using the MTS assay after 24–72 h post‐transfection. (B) Caspase‐3/7 activities were assessed for Fadu and SCC‐9 cells. The cells were transfected with siRNA1 (40 nM) for PD‐L1 or control siRNA, and the activities of caspases were measured 24 and 48 h post‐transfection. The data are plotted as the average relative activity. (C and D) Representative images and quantification of the reduction in migratory capacity (top panel) and invasion (bottom panel) of Fadu and SCC‐9 cells that were transfected with 40 nM of siRNA1 or siRNA2 for PD‐L1, compared to negative scramble controls. All the data were generated from three independent experiments. *p < 0.05; **p < 0.01.

The biological significance of PD‐L1 in HNSCC cells was further assessed. The cytotoxicity effects on transfected cells were estimated by the MTS assay. The reduction of PD‐L1 resulted in a significant decrease in cell viability (Figure 2A, *p* < 0.05). The silencing of PD‐L1 was also associated with activation of caspase‐3/7, and a significant increase in the caspases' activities was observed in PD‐L1‐expressing tumour cells 48 h after transfection (Figure 2B, *p* < 0.05), indicating that the stages of apoptosis are associated with changes in caspase activity. Furthermore, cell migration and cell invasion experiments demonstrated that knockdown of PD‐L1 resulted in a significant reduction in cell migration (average of 48% for both siRNAs) and invasion (55% for both) of Fadu cells compared to that of the scramble control (Figure 2C, *p* < 0.05). Similar reductions in cell migration and invasion were also observed in SCC‐9 cells (Figure 2D).

### 3.3 Interferon‐γ (IFN‐γ) induces PD‐L1 expression in HNSCC

**FIGURE 3:**
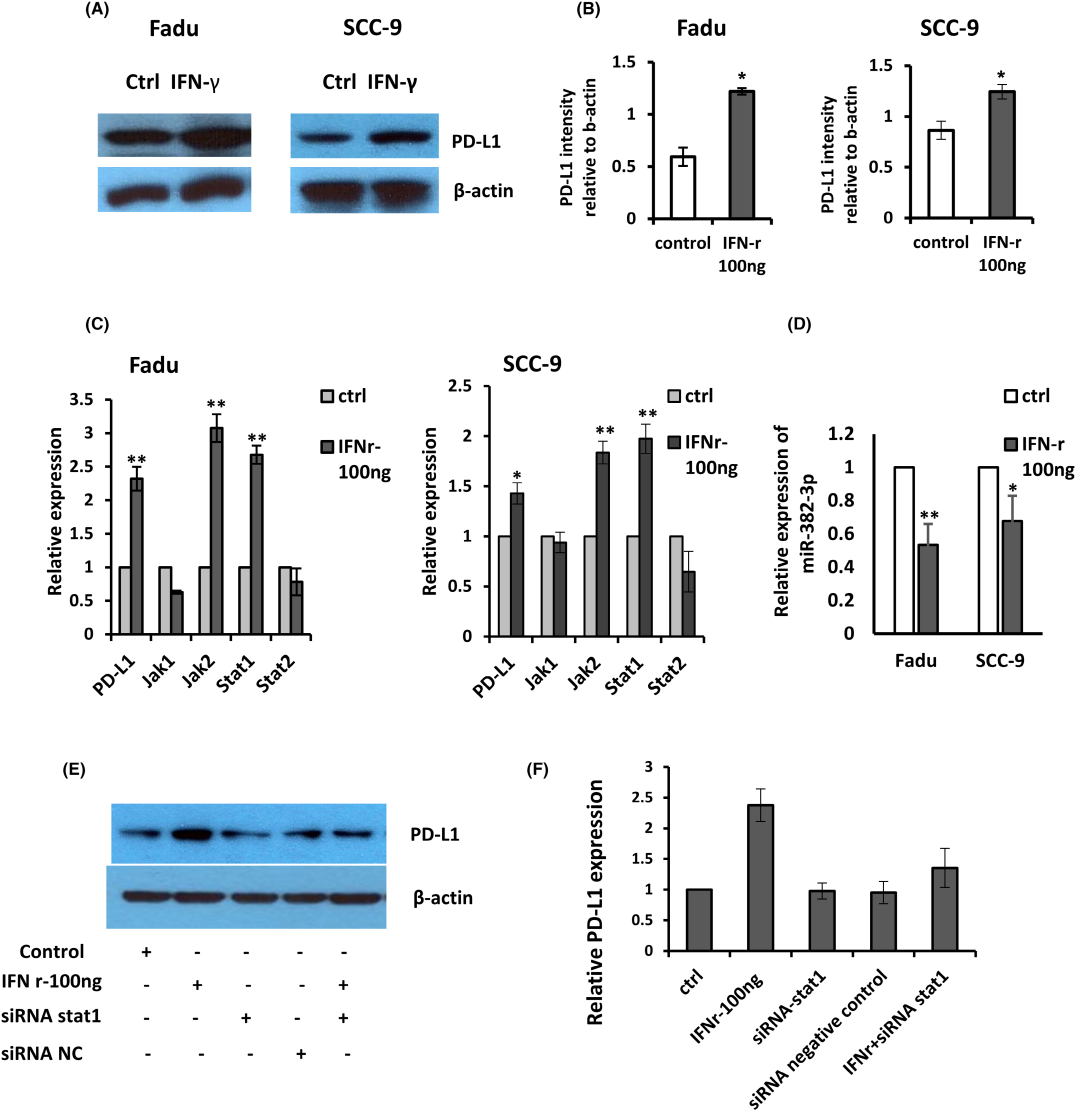
Interferon‐γ (IFN‐γ) induces PD‐L1 expression in HNSCC. (A and B) PD‐L1 expression levels were measured in Fadu and SCC‐9 cell lines 72 h after treatment with or without IFN‐ γ (100 ng). (C) JAK/STAT signalling pathway gene expression levels in cell lines 48 h after control or 100 ng IFN‐γ treatment. The levels of gene expression are normalized to β‐actin and GAPDH. (D) Expression of miR‐382‐3p in cell lines following IFN‐γ treatment. (E and F) Expression of PD‐L1 in Fadu cells was stimulated by IFN‐γ and Stat1. Each experiment was repeated at least three times. *p < 0.05, **p < 0.01.

The phenotypic findings discussed above implicate PD‐L1 involving in the malignancy of HNSCC. However, the underlining mechanism of PD‐L1 overexpression in HNSCC remains unclear. We first looked at how IFN‐γ affected PD‐L1 expression. The results showed that 100 ng of IFN‐γ strongly stimulated PD‐L1 protein and mRNA expression (Figure 3A,B). Subsequent qRT‐PCR demonstrated that Jak2 and Stat1 levels were increased remarkably, but not Jak1 and Stat2 (Figure 3C). IFN‐γ (100 ng) treatment, on the contrary, significantly reduced the expression of miR‐382‐3p (Figure 3D), but had no effect on the expression of circ_0000052 (Figure S2). Furthermore, while Stat1 (siRNA) depletion reduced PD‐L1 expression, the increase in PD‐L1 expression caused by IFN‐γ stimulation was reduced by the additional blockade of Stat1 (Figure 3E,F), indicating that Stat1 plays an important role in the effect of IFN‐γ on PD‐L1. These observations collectively revealed that IFN‐γ induces upregulation of PD‐L1 expression in HNSCC cells, one of its transcriptional mediators through activating of the Jak2 and Stat1 signal transducers.

### 3.4 miR‐382‐3p directly targets PD‐L1

**FIGURE 4:**
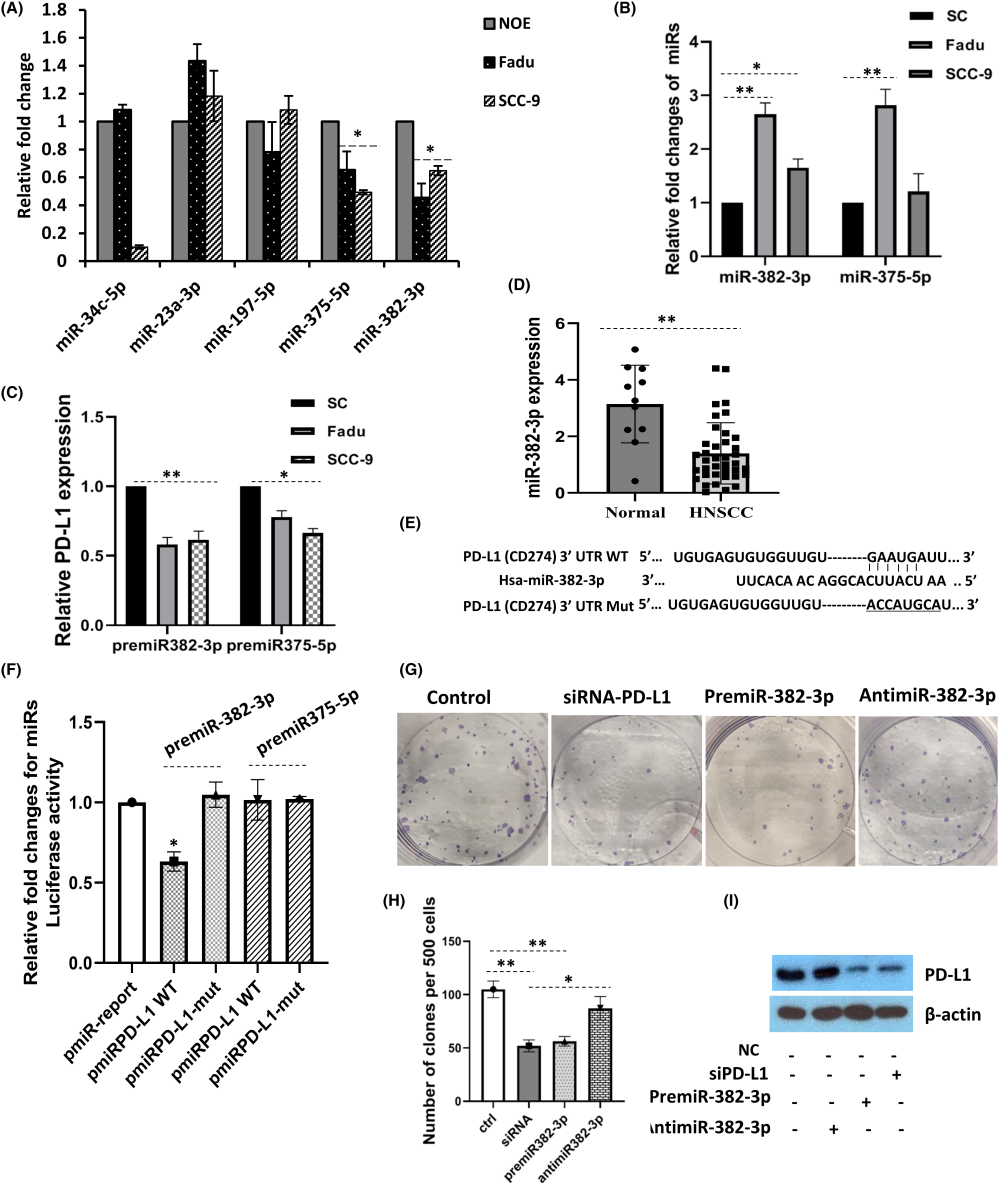
miR‐382‐3p directly targets PD‐L1 in HNSCC. (A) qRT‐PCR validation of five putative miRNA targets for PD‐L1 identified through bioinformatics analysis. (B) Mimics of miRNAs increased the expression of miR‐382‐3p and miR‐375‐5p in Fadu and SCC‐9 cells at 48 h post‐transfection. (C) PD‐L1 expression was reduced by transfection of premiR‐382‐3p and premiR‐375‐5p. (D) MiR‐382‐3p expression levels in HNSCC tissues were measured. (E) The targeting sites of the wide type (WT) or mutation (mut) of PD‐L1 with miR‐382‐3p. (F) Luciferase reporter assays in Fadu and SCC‐9 cells co‐transfected with pMIR‐PD‐L1 or pMIR‐PD‐L1‐mut plasmids, scramble negative control miR, and either premiR‐382‐3p or premiR‐375‐5p. Samples were analysed 72 h post‐transfection, and data were normalized to the pMIR report only transfection. (G and H) Colony formation assay of Fadu cells transfected with siRNA‐PD‐L1, premiR‐382‐3p or antimiR‐382‐3p. (I) PD‐L1 expression levels were regulated by miR‐382‐3p, as detected by Western blotting. Data represent the mean ± SE, *n* = 3. *p < 0.05, **p < 0.01.

MicroRNAs are powerful mediators in the post‐transcriptional regulation of gene expression. By utilizing gene target prediction softwares (miRWalk, miRDB, miRSystem, miRSearch and MicroT‐CDS) and combining various biological effects on tumour progression and cellular processes, five potential tumour suppressor miRNAs, which may be potential targets of PD‐L1, were selected for further study. qRT‐PCR was performed to access the cellular expression of each miRNA, and the results indicated that miR‐375‐5p and miR‐382‐3p were downregulated in Fadu and SCC‐9 cells compared with NOE cells (Figure 4A). Mimics of miR‐375‐5p (premiR‐375‐5p) and miR‐382‐3p (premiR‐382‐3p) effectively increased the expression of both miRNAs (Figure 4B), resulting in downregulation of PD‐L1 in both cells (Figure 4C), specifically by introducing mimics of miR‐382‐3p. MiR‐382‐3p expression was further determined in 38 available HNSCC tissues. The results showed that miR‐382‐3p was downregulated in the HNSCC compared to the normal adjacent tissues (Figure 4D). To investigate the direct interaction between miRNAs and PD‐L1, we constructed several reporter vectors carrying the predicted binding site(s) downstream of the firefly *luciferase* gene in the pMIR‐report vector as previously described.^16^ The overlapping seed site of miR‐382‐3p or miR‐375‐5p (Figure S3) with PD‐L1 was illustrated in Figure 4E. Fadu cells were co‐transfected with premiR‐382‐3p (or premiR‐375‐5p) and pmiR‐PD‐L1‐3' UTR (or a mutated pmiR‐PD‐L1‐3' UTR). By comparing with the control, the dual‐luciferase reporter assay showed that overexpression of miR‐382‐3p reduced luciferase activity by 40% in cells transfected with wide‐type PD‐L1 3′‐UTR, whereas the mutated reporter was unaffected (Figure 4F). In contrast, no effect on the luciferase activity was observed in cells overexpressing miR‐375‐5p, indicating a direct interaction between miR‐382‐3p and the 3'‐UTR of PD‐L1. To assess the relationship of miR‐382‐3p with PD‐L1 on cellular phenotype, colony formation assays were performed. The results showed that Fadu cells transfected with siPD‐L1 or premiR‐382‐3p displayed a significantly reduction of colony formation than that in the SC control. To functionally confirm the regulatory role between miR‐382‐3p and PD‐L1, we also carried out experiments by transfecting antimiR‐382‐3p in cells. As expected, the colony‐forming activity of the cells was not significantly affected (Figure 4G,H). Subsequent Western blots further confirmed the observations, where transfection of siPD‐L1 or premiR‐382‐3p led to a significant reduction in PD‐L1 expression levels but not for antimiR‐382‐3p (Figure 4I).

### 3.5 Circ_0000052 regulates miR‐382‐3p to affect proliferation, migration, and invasion

**FIGURE 5:**
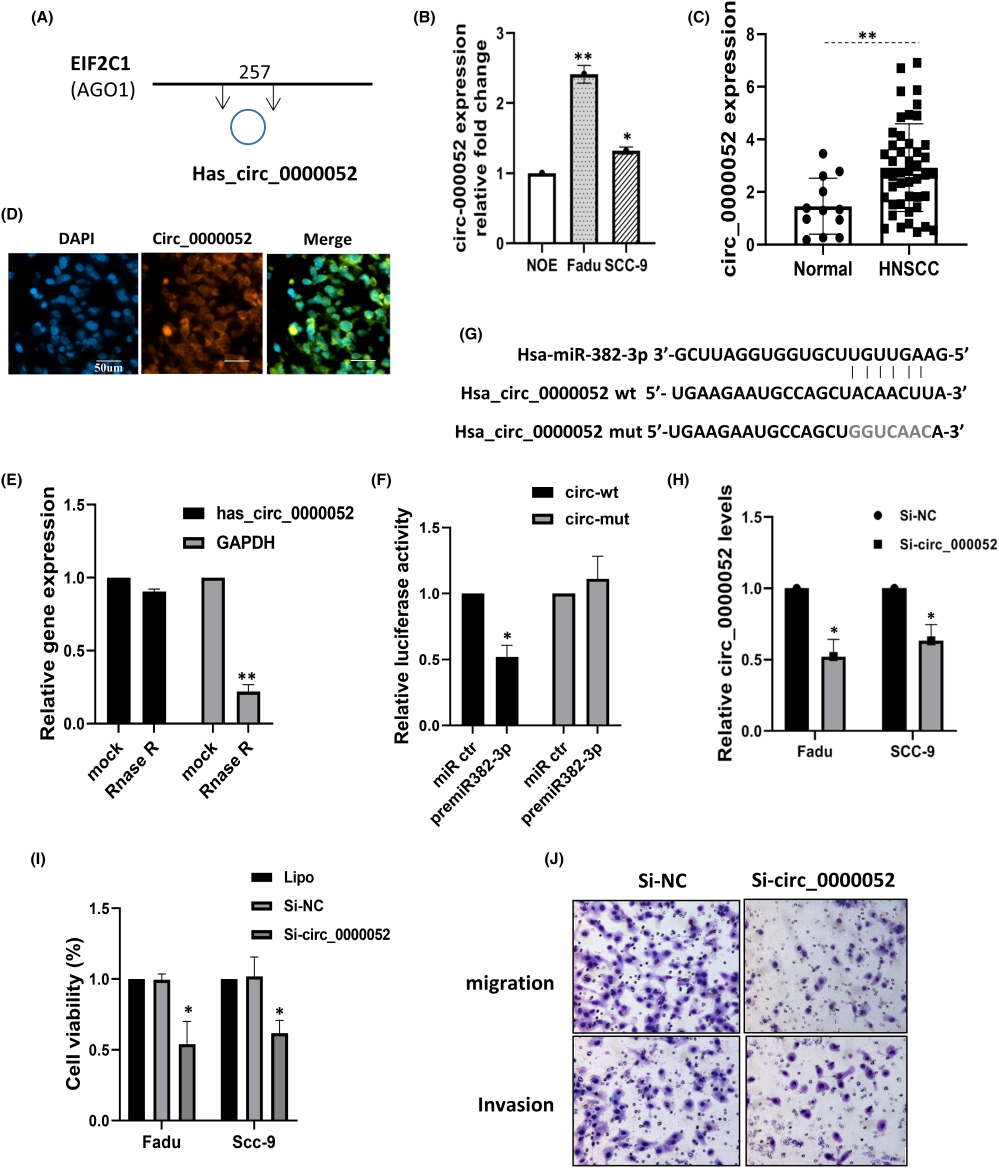
Circ_0000052 regulates cell growth and invasion by targeting miR‐382‐3p. (A) A schematic representation of circ_0000052 and its optimally transcribed gene. (B and C) The expression level of circ_0000052 was detected in HNSCC cells and tissue specimens. (D) The cellular distribution of circ_0000052 was analysed by fluorescence in situ hybridization (FISH). Nuclei were stained with DAPI (blue). Orange indicates circ_0000052. Scale bar = 50 μm. (E) The stability of circ_0000052 was determined by qRT‐PCR after treatment with RNase R or mock in total RNAs derived from Fadu cells. (F) Luciferase activity in Fadu cells co‐transfected with luciferase reporters containing miR‐382‐3p sequences with wild‐type or mutated circ_0000052 binding sites and the mimics of miR‐382‐3p or control. (G) Schema of the predicted binding site of miR‐382‐3p on the wide‐type (WT) of circ_0000052. A mutant sequence of circ_0000052 was shown. (H‐J) Depletion of circ_0000052 reduced cell viability, migratory capacity and cell invasion. All data were represented as means ± SE, *n* = 3. *p < 0.05; **p < 0.01.

Given the evidence that circRNAs can act as ceRNAs to regulate miRNA activity, we then investigated the possible relationship between circRNAs and miR‐382‐3p. By exploiting TargetscanHuman 7.2 and circular RNA interactome, the top 5 upregulated circRNAs were ranked based on the number of binding sites and the context score. Combined with the KEGG functional pathway analysis of HNSCC, circ_0000052 (circ_AGO1) was chosen as a candidate circRNA for study (Figure 5A). The expression levels of circ_0000052 in cell lines and available HNSCC tissue samples were detected by qRT‐PCR. The results demonstrated that the expression levels of circ_0000052 in Fadu and SCC‐9 cells as well as in HNSCC tissues were significantly higher than those in normal controls (Figure 5B,C). Immunofluorescence in situ hybridization indicated that circ_0000052 was distributed in the cytoplasm of Fadu cells (Figure 5D). The stability of circ_0000052 was analysed by RNase R treatment. qRT‐PCR results showed that linear RNA (GAPDH) was significantly reduced, but circ_0000052 was resistant and more stable to RNase R (Figure 5E). Subsequently, we treated cells with a transcription inhibitor, actinomycin D, and circ_0000052 was extremely stable compared to the linear mRNA (AGO1) (Figure S4). To verify the binding capability of miR‐382‐3p to circ_0000052, we constructed the wide and mutant‐type circ_0000052 luciferase reporter system and introduced miR‐382‐3p mimics or scramble RNA into Fadu cells. The results revealed that miR‐382‐3p significantly reduced luciferase activity on wide‐type circ_0000052, while it had no effect on the mutated vector (Figure 5F). The binding sites between circ_0000052 and miR‐382‐3p are depicted in Figure 5G. The pathologic role of circ_0000052 was further investigated. The overexpression of circ_0000052 was successfully knocked down by siRNA for circ_0000052 in Fadu and SCC‐9 cells (Figure 5H). This depletion resulted in decreased cell viability (Figure 5I) and reduced cell migration and invasion abilities (*p* < 0.05) (Figure 5J). Taking together, these data suggest that circ_0000052 promotes tumour progression in HNSCC.

### 3.6 Circ_0000052 interacts with PD‐L1 via sponge miR‐382‐3p

**FIGURE 6:**
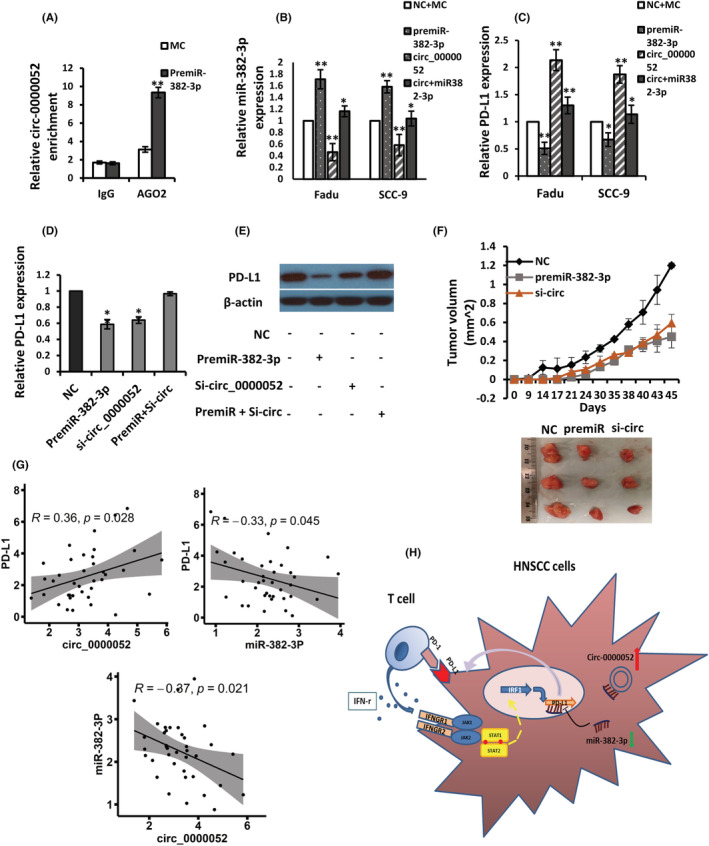
Circ_0000052 interacts with PD‐L1 via sponge miR‐382‐3p. (A) The RIP assay was executed in Fadu cells after introducing enriched miR‐382‐3p, followed by detecting the expression of circ_0000052. (B and C) After co‐transfection of cells with premiR‐382‐3p or overexpressed circ_0000052 plasmids or both, the expression levels of miR‐382‐3p or PD‐L1 were determined. (D and E) Rescue experiments were performed by transfecting premiR‐382‐3p or si‐circ_0000052 or co‐transfecting both. PD‐L1 levels were detected by qRT‐PCR and Western blotting. (F) Enhanced miR‐382‐3p and depletion of circ_0000052 delayed tumour growth compared with the control. *n* = 3/group. Tumour volume was measured. (G) The correlation between PD‐L1 and circ_0000052, PD‐L1 and miR‐382‐3p, circ_0000052 and miR‐382‐3p was analysed by Pearson correlation analysis. *p < 0.05, **p < 0.01. (H) A schematic diagram to illustrate a hypothetical model in which circ_0000052 acts as a competitive ceRNA to sponge‐adsorb miR‐382‐3p and alleviate the inhibitory effect on PD‐L1 through a circRNA‐sponge mechanism.

To further determine whether miR‐382‐3p and circ_0000052 could regulate PD‐L1 expression in HNSCC cells, premiR‐382‐3p was transfected into Fadu cells. RIP analysis demonstrated that anti‐Ago2 greatly pulled down circ_0000052 compared to the anti‐IgG control. Especially when cells were transfected with premiR‐382‐3p, circ_0000052 was more abundant than cells transfected with MC (Figure 6A) (*p* < 0.01). Subsequent transfection of premiR‐382‐3p and overexpressed circ_0000052 plasmids into Fadu and SCC‐9 cells showed that the expression of miR‐382‐3p was significantly increased by introducing a mimic of miR‐382‐3p, but was downregulated by circ_0000052. However, co‐transfection of both premiR‐382‐3p and circ_0000052 plasmids rescued this change when compared with the NC + MC control (Figure 6B, *p* < 0.01). Notably, the expression levels of PD‐L1 were reduced by premiR‐382‐3p, while the circ_0000052 plasmid significantly increased the expression level of PD‐L1. The co‐transfection of premiR‐382‐3p and circ_0000052 plasmids could balance out the increased PD‐L1 expression (Figure 6C, p < 0.01). In addition, when the cells were introduced with premiR‐382‐3p or si‐circ_0000052, or both, the results indicated that PD‐L1 expression was significantly decreased in miR‐382‐3p overexpressing cells and circ_0000052 knockdown cells compared with negative control. However, PD‐L1 expression was rescued by co‐transfection with both premiR‐382‐3p and si‐circ_0000052 (Figure 6D,E). Moreover, after transfection of the lentiviruses containing sh‐circ_0000052 vector or premiR‐382‐3p, or NC into Fadu cells, tumour formation experiments demonstrated that both sh‐circ_0000052 and premiR‐382‐3p inhibited tumour growth obviously in comparison with the NC group (*p* = 0.042, Figure 6F).

Finally, the correlation between circ_0000052, miR‐383‐3p or PD‐L1 was analysed by Pearson's correlation coefficient method in HNSCC tissues. The results depicted that the expression of circ_0000052 was positively correlated with PD‐L1 level (*R* = 0.36, *p* = 0.028), while the level of miR‐383‐3p was correlated negatively with both circ_0000052 (*R* = −0.37, *p* = 0.021) and PD‐L1 mRNA (*R* = −0.33, *p* = 0.045) (Figure 6G). Putting the data together, they implicate that circ_0000052 could enhance PD‐L1 expression by sponge‐adsorbing miR‐382‐3p in HNSCC cancer cells, as illustrated in Figure 6H.

## 4 DISCUSSION

A major challenge in the current treatment of HNSCC, especially in advanced patients, is the need to improve treatment strategies and develop prognostic indicators. Despite the great success of immune checkpoint blockade therapy with PD‐1/PD‐L1 in the treatment of many solid malignancies, a large proportion of HNSCC patients remain unresponsive, including HNSCC.^17^, ^18^ As a result, gaining a better understanding of the mechanisms underlying PD‐L1 aberrant expression in HNSCC will aid in the discovery of potential predictive tumour biomarkers and the overcoming of treatment resistance. In the current study, we provide evidence that PD‐L1 is highly expressed in HNSCC samples and cells. The PD‐L1 expression level was significantly correlated with patient survival. Depletion of PD‐L1 decreased tumour cell proliferation, migration, invasion and increased cell apoptosis.

The expression of PD‐L1 in tumours is regulated by multiple factors. Despite accumulating evidence documenting that PD‐L1 is overexpressed in human malignancies, the exact mechanisms that lead to PD‐L1 overexpression in cancer development and progression remain largely unknown. Tumour cells are generally thought to express PD‐L1 in order to protect them from immune elimination through two mechanisms: innate and adaptive immune resistance.^19^ During antitumor immune responses, PD‐L1 expression is induced mainly by inflammatory factors secreted primarily by a heterogeneous group of immune cells, which is known as adaptive immunity. Interferon gamma (IFN‐γ) is widely regarded as the most important T cell‐derived cytokine and plays an important role in immune regulation. IFN‐γ recruits and translocates STAT into the nucleus by binding to the interferon gamma receptor 1/2 (IFNGR1/2), active JAK1 and JAK2, resulting in target gene expression.^20^ Studies report that the transcription factor JAKs/STATs axis primarily regulates PD‐L1 expression, while JAK2 seems to play a dispensable role in melanoma.^21^ siRNA knockdown of interferon regulatory factor‐1 (IRF‐1) is responsible for the IFN‐gamma‐mediated PD‐L1 upregulation in human lung cancer cell lines.^22^ In addition, tyrosine kinase phosphorylation is mediated by EGFR mutation, which in turn activates NF‐κB, resulting in PD‐L1 overexpression in lung cancer.^23^ Furthermore, EBV‐induced latent membrane protein 1 (LMP1) upregulates PD‐L1 in nasopharyngeal carcinoma (NPC) through STAT3, AP‐1 and NF‐κB pathways.^24^ In our study, we show that IFN‐γ signalling induces PD‐L1 expression in HNSCC cells via the transcription factor Jak2 and Stat1. Unlike in melanoma,^21^ our results highlight the important role of Jak2 and Stat1 in inducing PD‐L1 expression in HNSCC. Furthermore, our data suggested that interferon gamma can downregulate the expression of miR‐382‐3p but not circ_0000052. The underlying mechanism requires further elucidation.

MicroRNAs mediate tumour development and progression by suppressing target mRNA translation. PD‐L1 3'‐UTR can be directly targeted by several microRNAs. MiR‐152 expression was significantly decreased in human gastric cancers. Since miR‐152 is directly bound to the PD‐L1 3'‐UTR, a mimic of miR‐152 suppressed PD‐L1 expression.^25^ Furthermore, in HNSCC, down regulation of the let‐7 family was negatively correlated with PD‐L1 expression.^11^ By using bioinformatics tools, miR‐382‐3p and miR‐375‐5p were identified as consistently underexpressed in HNSCC cells and had potential binding sites on PD‐L1. Subsequent luciferase reporter assays revealed that miR‐382‐3p could directly target PD‐L1. Moreover, mimicking increased miR‐382‐3p could reduce PD‐L1 expression, thereby reducing colony formation. MiR‐382 was found to be downregulated and has the ability to directly target the oncogene LIM only protein 3 (LMO3).^26^ From peer‐reviewed studies, miR‐382‐3p was found to be a microRNA capable of inhibiting tumour PD‐L1 in colorectal cancer cells and significantly reducing tumour migration, and stimulating anti‐tumour immune responses.^27^ It is worth noting that miR‐382‐3p was also underexpressed in our HNSCC tissues. Our findings fully demonstrate an alternate mechanism, in which miR‐382‐3p can directly target PD‐L1, and deletion of miR‐382‐3p releases PD‐L1 repression in HNSCC.

CircRNA is an endogenous non‐coding RNA. It is more stable than its linear counterpart due to the absence of a 5′ cap and 3′ polyadenylation tails.^28^ Current studies have shown that competing endogenous RNA (ceRNA) can regulate each other's expression by competitively binding miRNAs, thereby regulating their downregulated target genes.^29^ As an important member of the ceRNA network, one of the main functions of circRNAs is to imbibe miRNAs, thereby regulating target mRNAs. The reason for the downregulation of miR‐382‐3p remains elusive, with only one recent study showing that lncRNA PMSB8‐AS1 can directly bind miR‐382‐3p, resulting in its downregulation in pancreatic cancer.^30^ In the current study, by combining bioinformatics prediction and transcriptomic analysis, we found that circ_0000052 has a putative binding site for miR‐382‐3p. Dual‐luciferase reporter combined with RIP assays indicated a direct negative interaction between circ_0000052 and miR‐382‐3p. Circ_0000052 is located at chr1:36367548‐36367938. Its best transcript has been identified as argonaute RISC component 1 (AGO1) (https://www.ncbi.nlm.nih.gov/nuccore/NM_012199). This gene encodes a member of the argonaute protein family and plays a central role in the regulation of RNA interference and RNA silencing.^31^ Studies have shown that AGO1 is highly expressed tissue‐specifically in lung cancer,^32^ colon cancer^33^ and breast cancer,^34^ especially with disease progression. However, the functions of circ_0000052 in HNSCC are unknown. Our data revealed that circ_0000052 was upregulated in clinical tissue specimens and HNSCC cells, and the expression of circ_0000052 in corresponding samples was positively correlated with the expression level of PD‐L1. Furthermore, knockdown of circ_0000052 inhibited HNSCC cell growth, mobility and tumorigenesis. These data imply a carcinogenic role for circ_0000052 in HNSCC progression. Combined with our previous findings, there is a direct targeting relationship between PD‐L1 and miR‐382‐3p. Through different rescue experiments by enhancing miR‐382‐3p, or enhancing or depleting circ_0000052 in vitro or in vivo, we show that circ_0000052 can competitively bind with miR‐382‐3p and reduce the expression of miR‐382‐3p, thereby releasing the inhibitory effect of miR‐382‐3p on PD‐L1, leading to the upregulation of PD‐L1 in HNSCC cells. Our current data reveal a novel mechanism by which circ_0000052 can promote tumour progression by regulating PD‐L1 expression through sponging miR‐382‐3p in HNSCC cells, as illustrated in Figure 6H. To the best of our knowledge, this is the first report describing the function of circ_0000052 and linking the regulatory role of the circ_0000052/miR‐382‐3p/PD‐L1 axis in HNSCC.

In conclusion, our data show that overexpression of PD‐L1 is pivotal at the clinical and basic cellular levels to regulate cell proliferation, migration, invasion and clonogenicity in HNSCC. IFN‐γ/JAK2/STAT1 signalling plays an important role in the induction of PD‐L1 overexpression. Furthermore, a novel mechanism has been demonstrated, whereby upregulated circ_0000052 mediates PD‐L1 overexpression by competitively binding to miR‐382‐3p and alleviating the inhibitory effect on PD‐L1, leading to overexpression of PD‐L1. These findings indicate that the circ_0000052/miR‐382‐3p/PD‐L1 axis may be a candidate for molecularly targeted therapy in HNSCC.

## AUTHOR CONTRIBUTIONS

**De‐Jun Zhang:** Data curation (equal); formal analysis (equal); investigation (equal); methodology (equal); project administration (equal); software (equal); validation (equal); writing – original draft (equal); writing – review and editing (equal). **Ze‐Ming Fu:** Data curation (equal); formal analysis (equal); investigation (equal); methodology (equal); project administration (equal); software (equal); validation (equal); writing – original draft (equal); writing – review and editing (equal). **Ying‐Yuan Guo:** Project administration (equal); resources (equal); validation (equal); writing – review and editing (equal). **Fang Guo:** Project administration (equal); resources (equal); software (equal); writing – review and editing (equal). **Yi‐Ning Wan:** Project administration (equal); resources (equal); software (equal); writing – review and editing (equal). **Guofang Guan:** Conceptualization (equal); funding acquisition (equal); investigation (equal); resources (equal); visualization (equal); writing – review and editing (equal).

## CONFLICTS OF INTEREST

The authors declare no conflict of interest.

## Supporting information


**Data S1:** Supporting Information

## Data Availability

Datasets are available from the corresponding author upon reason‐able request.
