# Supplementary material for: Circ_0000052/miR‐382‐3p axis induces PD‐L1 expression and regulates cell proliferation and immune evasion in head and neck squamous cell carcinoma
Source: J Cell Mol Med. 2022 Dec 14;27(1):113–26. doi: 10.1111/jcmm.17643 (PMC9806294; doi:10.1111/jcmm.17643)
Supplement: Supplementary file 1 — Data S1: Supporting Information [file JCMM-27-113-s001.pdf]

Circ_0000052/miR-382-3p axis induces PD-L1 expression and regulates cell proliferation and immune evasion in head and neck squamous cell carcinoma

Supplementary:

Supplementary Table 1. Primer sequences

Gene		Primer sequence
PD-L1	For	5'- GTGGCATCCAAGATACAAACTCAA -3'
	Rev	5'- TCCTTCCTCTTGTCACGCTCA -3'
GAPHD	For	5'- GTCTCCTCTGACTTCAACAGCG-3'
	Rev	5'- ACCACCCTGTTGCTGTAGCCAA-3'
PD-L1-WT	For	5'-TGTTGGAACGGGACAGTATTT-3'
PD-L1-WT	Rev	5'-TGAGCAAATCATTAAGCAGCA-3'
PD-L1-mut	For	5'-CATCCTAGGAAGACGGGTTG-3'
PD-L1-mut	Rev	5'-CAACCACACTCACATGACAAGA-3'
JAK1	For	5'- AGCGATGTCCTTACCACACC -3'
	Rev	5'- CCTCAACACACTCAGGAGCA -3'
JAK2	For	5'- GAGCCTATCGGCATGGAATA -3'
	Rev	5'- ACTGCCATCCCAAGACATTC -3'
STAT1	For	5'- CCGTTTTCATGACCTCCTGT -3'
	Rev	5'- TGAATATTCCCCGACTGAGC -3'
STAT2	For	5'-CAGGTCACAGAGTTGCTACAGC-3'
	Rev	5'-CGGTGAACTTGCTGCCAGTCTT-3'
circ-0000052	For	5'- GACAACCAGACCTCGACCAT -3'
	Rev	5'- CTCCGTCATGTCATCCTTCA -3'
linear-0000052 (AGO1)	For	5'- ACAAGTCCACCCGTTTCAAG -3'
	Rev	5'- CCAGCTGGGATGTTACCACT -3'
circ-0000052-WT	For	5'- GACAACCAGACCTCGACCAT -3'
circ-0000052-WT	Rev	5'- CTCCGTCATGTCATCCTTCA -3'
circ-0000052-mut	For	5'- CATCTGCCCTGCCTACAAGT -3'
circ-0000052-mut	Rev	5'- GCGACTGATCTCCTCCTGTC -3'
si-circ-0000052		5'-GAGAAGAGGTGCTCAAATGAA-3'

Supplementary Figure 1.

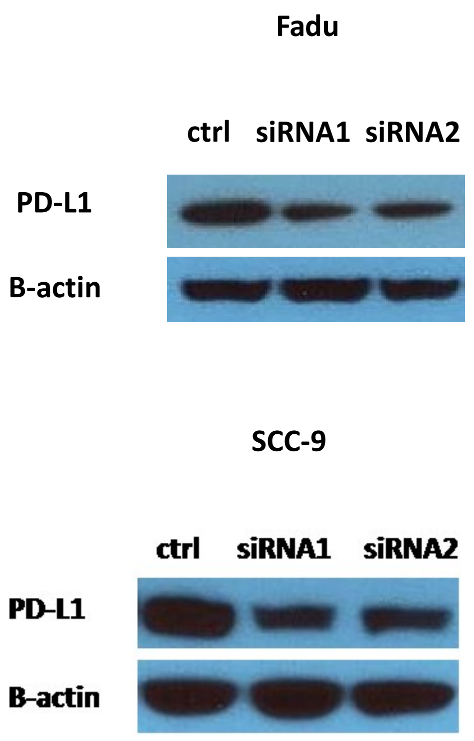

PD-L1 depleted effectively by siRNAs examined by Western blot.

Supplementary Figure 2.

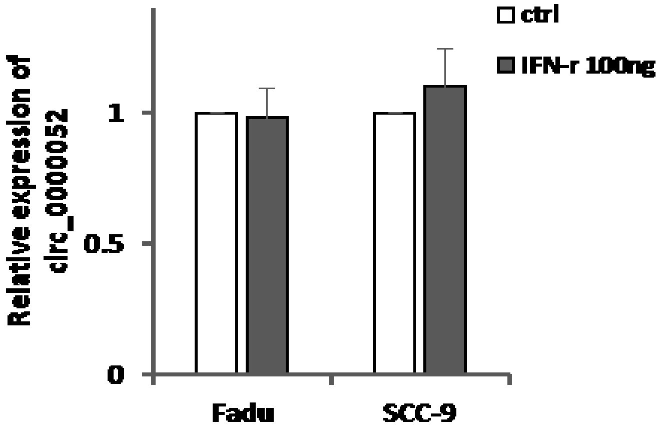

HNSCC cells treated with IFN-γ (100ng) had no effects on the expression of circ_0000052.

Supplementary Figure 3.

```
PD-L1 (CD274) 3’ UTR WT     5’ ….. GCTCTAGGAC AGA…….GTTTGGA TTT… 3’
                                                     ||||||
    hsa-miR-375-5p             3’…..    GCGACGAGCC… GCACAAACC…5’
PD-L1 (CD274) 3’ UTR MUT 5’ ….. GCTCTAGGAC AGA…….TAGCGCAAT… 3’
```

The potential targeting sites of wide type (WT) or mutation (mut) of PD-L1 with miR-375-5p.

Supplementary Figure 4.

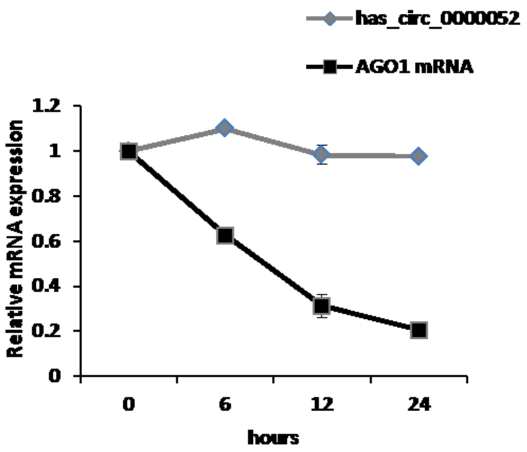

The relative mRNA levels were measured by qRT-PCR after treatment with actinomycin D. Samples were collected at indicated time points in Fadu cells.
